# Electroacupuncture at Baliao point alleviates post-operative pain and anal distension after procedure for prolapse and hemorrhoids (stapled hemorrhoidopexy): a prospective randomized clinical trial

**DOI:** 10.1007/s00384-023-04403-y

**Published:** 2023-04-19

**Authors:** Jiamen Shen, Xiecheng Zhou, Jiaying Zhao, Huipeng Wang, Tao Ye, Wenjie Chen, Xin Wang, Lifeng Gong, Yuankun Cai

**Affiliations:** Department of General Surgery, The Fifth People’s Hospital of Shanghai, Fudan University, No. 801 Heqing Road, Shanghai, 200240 People’s Republic of China

**Keywords:** Acupuncture, Acupuncture points, Hemorrhoids, Pain, Postoperative

## Abstract

**Purpose:**

The purpose of this study was to explore the effect of electroacupuncture (EA) at Baliao point on short-term complications, such as anal pain and swelling, after procedure for prolapse and hemorrhoids (PPH) in patients with mixed hemorrhoids.

**Methods:**

A total of 124 eligible patients undergoing PPH surgery were included in this study and randomly divided into a control group (*n* = 67) and an EA group (*n* = 57), with patients in the control group receiving only PPH surgery and patients in the EA group receiving PPH surgery and EA at Baliao point.

**Results:**

The visual analogue scale (VAS) scores of EA group at 8, 24, 48, and 72 h after operation were significantly lower than those of control group. The anal distension scores at 8, 48, and 72 h after operation were also significantly lower than those of control group. The number of postoperative analgesic drug administration per patient was also significantly lower in the EA group. The incidence of urinary retention and tenesmus in EA group was significantly lower than that in control group within the first day after surgery.

**Conclusion:**

EA treatment at the Baliao point can alleviate short-term anal pain and anal swelling after the procedure for prolapse and hemorrhoids, reduce the incidence of urinary retention, and decrease the use of postoperative analgesic drugs.

**Trial registration:**

This study was approved and registered by the Chinese Clinical Trial Center, Registration number: ChiCTR2100043519, Registration time: February 21, 2021 (https://www.chictr.org.cn/).

## Introduction

Mixed hemorrhoids are a common disease that afflicts people all over the world, and PPH is a common operation for the treatment of mixed hemorrhoids [1, 2]. However, it is difficult for patients to recover pelvic floor function in short term after PPH surgery. The incidence of short-term complications after PPH, such as postoperative pain, anal distension, and urinary retention, is more than 40% [3, 4]. Unfortunately, there is a lack of effective treatment for these short-term complications, which has a serious impact on postoperative psychology, recovery, and quality of life. It is a challenge for the anorectal community to effectively promote the recovery of patients after PPH for mixed hemorrhoids [5].

**Fig. 1:**
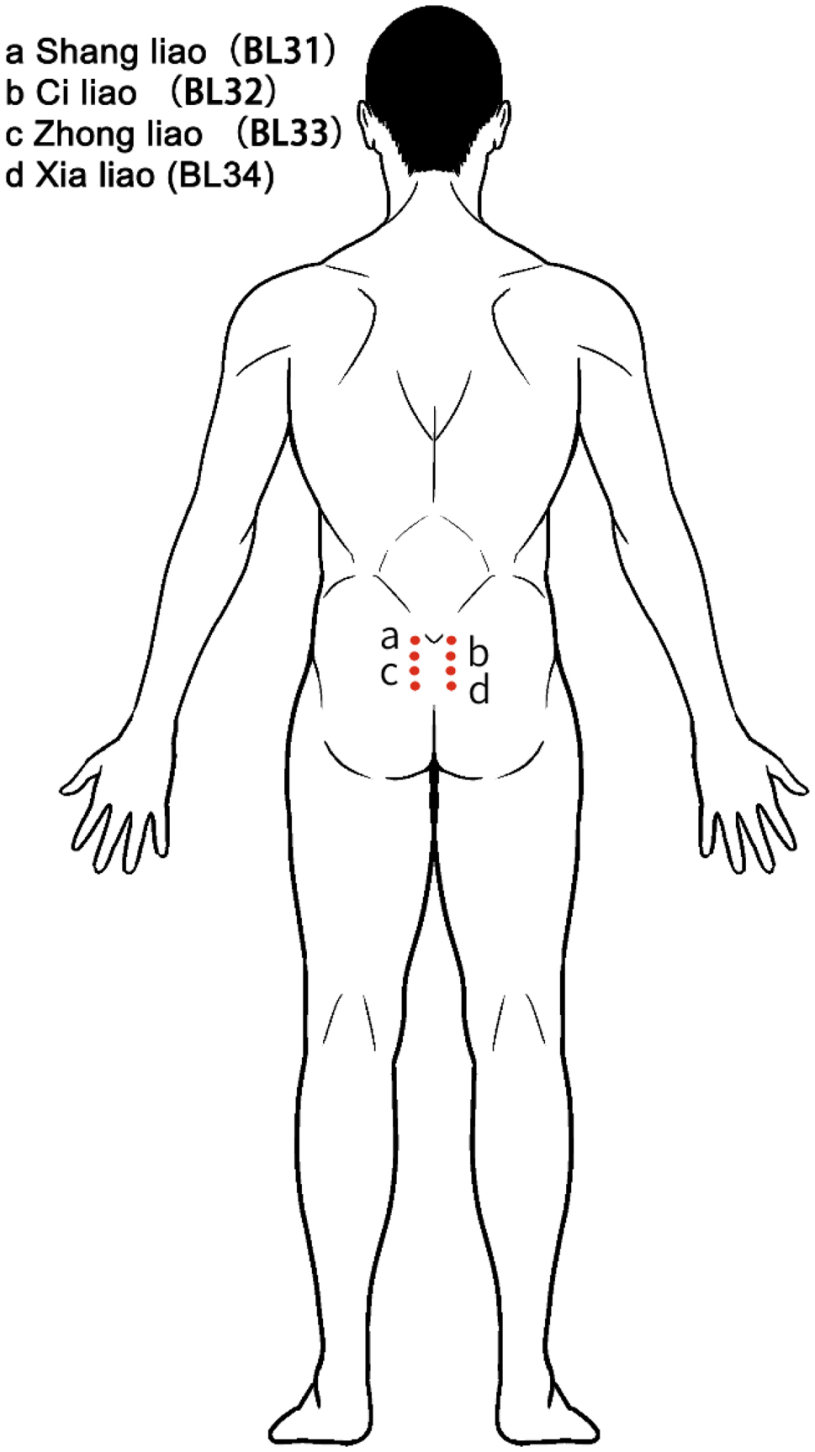
Anatomical position of Baliao point

The Baliao point (BL31–BL34) is a traditional Chinese acupuncture point with a long history. They are divided into four pairs, namely Shang liao (BL31), Ci liao (BL32), Zhong liao (BL33), and Xia liao (BL34), with a total of eight acupuncture points (Fig. 1) [6]. Acupuncture at Baliao point is often used in the treatment of gynecological, obstetric diseases, and pelvic floor diseases. It has a good effect on dysmenorrhea, functional defecation disorders, and stress urinary incontinence [7–9]. Recent studies have shown that the application of acupuncture Baliao point after pelvic floor surgery is helpful for postoperative analgesia after uterine curettage and anorectal surgery [10]. It has also been reported that acupuncture at Baliao point can promote the recovery of anorectal dynamics in postoperative patients, which is beneficial to the prognosis of patients undergoing anorectal surgery [11].

In China, some scholars have initially explored the effect of EA at Baliao point on anorectal diseases and postoperative recovery after anorectal surgery. It had been reported that acupuncture at Baliao point can promote anorectal motility and improve functional defecation disorders [9, 12]. The study of Long et al. showed that EA at Baliao point combined with auricular point sticking can treat anus pain after external hemorrhoids incision and internal ligation [11]. Song et al. reported that EA at Baliao point 30 min before operation can effectively reduce the degree of anal distension and pain at 6, 12, and 18 h post PPH operation for mixed hemorrhoids. It shorten the duration of anal distension and pain within 24 h after operation and shorten the time of first urination [13].

The purpose of this study is to further explore the effect of EA at Baliao point on the improvement of postoperative pelvic floor function in patients who underwent PPH surgery through a single disease in moderate sample size with more comprehensive observation indicators.

## Patients and methods

This study was an investigator blinded randomized pilot study. Since the lack of objective, prospective studies on the effects of EA at Baliao point on patients after PPH, the actual sample size calculations could not be performed. And this study was conducted in accordance with the ethical, moral, and scientific principles stipulated in the Helsinki Declaration and Guideline for good clinical practice. In addition, this study was approved and registered by the Chinese Clinical Trial Center, Registration number: ChiCTR2100043519, Registration time: February 21, 2021.

### Participants

**Fig. 2:**
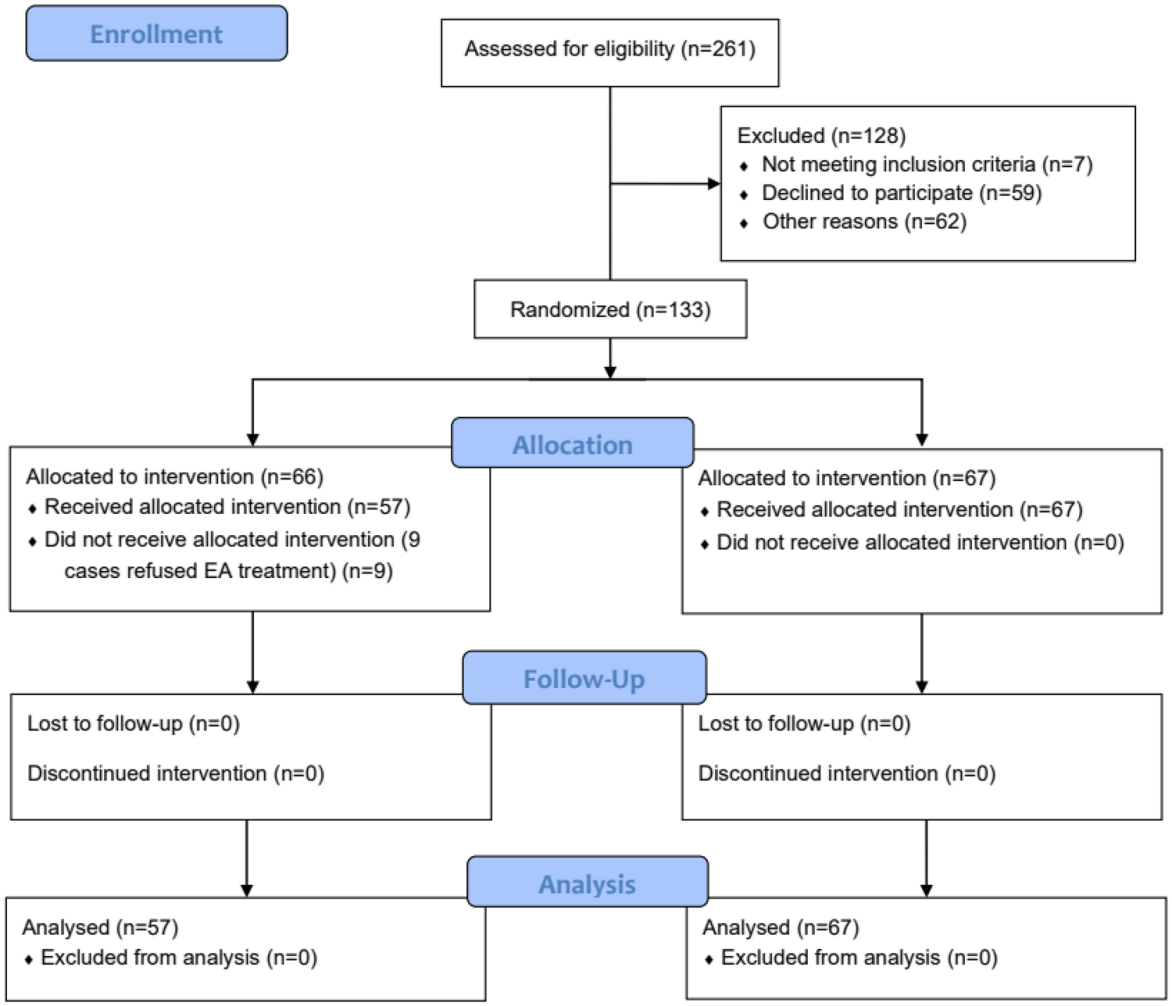
CONSORT 2010 flow diagram

A total of 261 patients with mixed hemorrhoids who underwent PPH surgery in Shanghai Fifth People’s Hospital from March 2021 to March 2022 were identified with 128 patients were excluded. The most common reason for exclusion was meeting our exclusion criteria. Those who met the exclusion criteria were 121 (Table 1), and 7 did not met the inclusion criteria. 133 cases were included in the study, divided into the EA group and the control group, the control group has 67 cases and the EA group has 66 cases according to a randomized numerical table method. However, the EA group had 9 cases refused EA treatment of Baliao acupoint; these 9 cases were excluded. Finally, EA group had 57 patients compared to 67 in the control group (Fig. 2).

**Table 1:** Reasons for patient exclusion

**Reason for exclusion**	***n*** **(%)**
Those who have poor compliance and do not accept this clinical trial	59 (48.76)
Combined with perianal infection and other benign anorectal diseases	20 (16.53)
Patients who require additional use of analgesia pump after surgery	15 (12.40)
Patients with prostatic hyperplasia, diabetes, and other underlying diseases that may affect the postoperative observation indexes	12 (9.92)
History of previous inguinal hernia surgery, urethral tension-free suspension surgery, and other surgery that may affect pelvic floor function	9 (7.44)
Pregnant women, menstrual, and lactating women	4 (3.31)
Patients with mental disorders	2 (1.65)

Total *N* = 121

#### Inclusion criteria

(1) patients with severe internal hemorrhoids or mixed hemorrhoids with surgical indications; (2) age between 18 and 70 years old; (3) no history of anorectal surgery; (4) patients gave informed consent to participate in this study.

#### Exclusion criteria

(1) patients with mental disorders; (2) those who have poor compliance and do not accept this clinical trial; (3) pregnant women, menstrual, and lactating women; (4) combined with perianal infection and other benign anorectal diseases; (5) patients with prostatic hyperplasia, diabetes, and other underlying diseases that may affect the postoperative observation indexes; (6) history of previous inguinal hernia surgery, urethral tension-free suspension surgery, and other surgery that may affect pelvic floor function; (7) patients who require additional use of analgesia pump after surgery.

### Randomization

Patients were randomized to receive either the control group or the EA group using the sealed envelope method by means of a one-to-one treatment allocation. The randomization was carried out by a clinician not involved in this study. Treatment assignments were generated using a randomized numerical table method. Envelopes were numbered sequentially, opaque. The outside serial number envelope was in line with the patient's visit sequence. The physician provided the enrolled patient with a sealed envelope labeled with the serial number. The patient then opened the sealed envelope containing the patient’s random group information. The primary investigator knew nothing about patient assignment until the study and data analysis were complete.

### Procedure

Preoperative preparation: All patients were given glycerol enema 2 h before surgery to empty the stool.

Surgical procedure of PPH: Spinal anesthesia was used for anesthesia. After successful anesthesia, the patient took the jackknife position. The surgical field was routinely disinfected with iodophor, and sterile towels and sheets were laid. The anal dilator was inserted into the anus to expose the hemorrhoids and dentate line. Loop suture of the rectal mucosa with a 2–0 absorbable suture at 3.0 cm from the dentate line. One stitch was sutured with silk in the direction of 3 o’clock and 9 o’clock of the anus. The stapler was inserted, and the mushroom head purse was ligated. The traction wire was drawn out from the side hole, tightened the stapler, held it for 1 min after firing, and slowly withdrew the stapler after loosening. Checked the anastomosis and sutured with 3–0 absorbable suture at the bleeding place to stop the bleeding. Vaseline gauze and hemostatic gauze were used to compress the wound, and the operation was completed.

Postoperative analgesic measures: After operation, ketorolac tromethamine 30 mg, q12h, was given intravenously for analgesia.

When the patient’s pain is significantly relieved, it will be discontinued. The dose will be increased if necessary. All increased doses of analgesics will be recorded.

Severe pain will be treated with an opioid (Bucinnazine Hydrochloride Injection).

Operation process of EA at Baliao point: The bilateral Ci liao (BL32) and bilateral Zhong liao (BL33) were selected as acupuncture points (a total of 4 acupuncture points on both sides). Alcohol cotton balls were used to disinfect the acupoints, and 0.30 × 75 mm needles were used for acupuncture with a depth of about 3 cm. The needle was connected to the CMNS6-1 needle stimulator, and EA was stimulated for 20 min (the setting conditions were continuous wave, 40 Hz, level 2 intensity). And the acupuncture therapies were performed by senior attending physicians of the Department of Traditional Chinese Medicine of the Fifth People's Hospital of Shanghai.

### Outcomes

Main outcome measures: The primary endpoint was the VAS scores and the anal distension scores at 8, 24, 48, and 72 h after operation. Secondary endpoints were urination, time to first defecation after surgery, feeling of tenesmus, use of postoperative analgesic drugs, and other related indexes which were observed after surgery.

Postoperative pain: VAS was used to evaluate the pain of surgical incision in patients at 8 h, 24 h, 48 h, and 72 h after the operation.

Postoperative anal distension: According to the following criteria, postoperative anal distension was divided into 4 grades, grade I (0 points): no anal distension; grade II (2 points): occasional seizures or mild symptoms; grade III (4 points): frequent, severe symptoms, affecting life, can be relieved after rest and treatment; grade IV (6 points): frequent, affecting daily life, and cannot be relieved even after rest and treatment.

Postoperative analgesic drug use: the administration times and additional use of ketorolac tromethamine were recorded for each patient. In addition, every use of opioid (Bucinnazine Hydrochloride Injection) should be recorded.

Urination 24 h after operation: The evaluation criteria were as follows: grade I (0 points): urination was unobstructed without urination disorder; grade II (2 points): slightly impaired urination, able to urinate without treatment; grade III (4 points): able to urinate on their own, but with less urine volume, abdominal distension and other related signs and symptoms, requiring conservative treatment; grade IV (6 points): no urine is excreted and catheterization is required.

Postoperative defecation: The patient’s first defecation time, daily defecation frequency, and feeling of urgency after surgery were recorded.

Adverse events occurred during the experiment, including but not limited to bleeding, infection, needle sickness, and unplanned reoperations which were recorded.

One month after the operation, the patients were followed by telephone to self-observe the following indicators: whether there was repeated anal pain; whether there was an anal bulge that was difficult to relieve; whether there was tenesmus; whether there was repeated constipation and diarrhea; and other adverse reactions or discomfort symptoms.

### Statistical analysis

Data were analyzed using GraphPad 9.0 software. Measurement data were expressed as mean ± standard deviation (*x* ± *s*). Independent samples *t* test was used to compare measurement data with normal distribution between groups, and chi-square test or Fisher’s exact test was used to compare count data. *P* < 0.05 was statistically significant.

## Results

### Subject demographics

From March 2021 to March 2022, a total of 124 patients with hemorrhoids who met the inclusion criteria and underwent PPH surgery were collected in Shanghai Fifth People’s Hospital. The patients were randomly assigned to the control group (*n* = 67) and the EA group (*n* = 57). The characteristics of the two groups of patients were similar, and no significant differences were found in age, sex, height, weight, or BMI (Table 2).

**Table 2:** Characteristics of the patients

Group	Control (*n* = 67)	EA (*n* = 57)	*P* value
Age, mean ± SD, y	47.22 ± 15.64	46.54 ± 13.10	> 0.05
Gender (male:female)	36: 31	26: 31	> 0.05
Height, mean ± SD, m	1.65 ± 0.09	1.66 ± 0.10	> 0.05
Weight, mean ± SD, kg	63.05 ± 11.24	64.89 ± 11.25	> 0.05
BMI, mean ± SD, kg/m^2^	23.09 ± 2.89	23.37 ± 3.19	> 0.05
Bristol Stool Scale, mean ± SD	3.96 ± 0.56	3.93 ± 0.62	> 0.05

### Primary outcomes

**Fig. 3:**
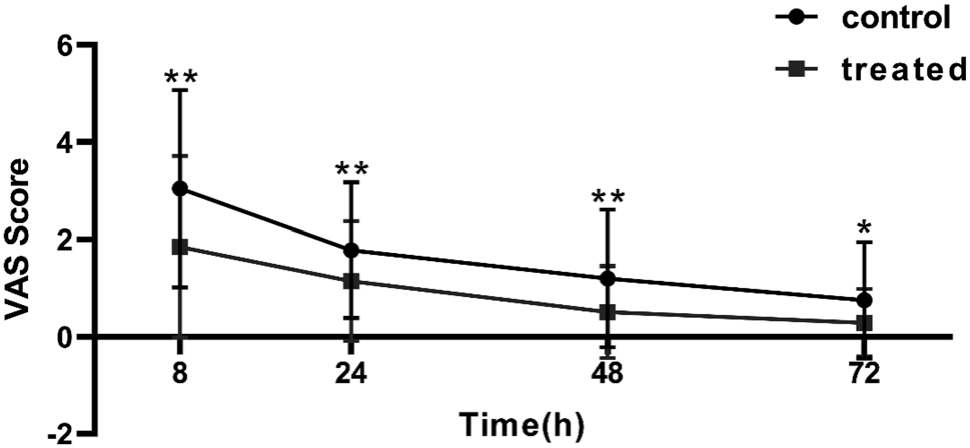
VAS pain score within 72 h after operation. Note: *P < 0.05, **P < 0.01

**Fig. 4:**
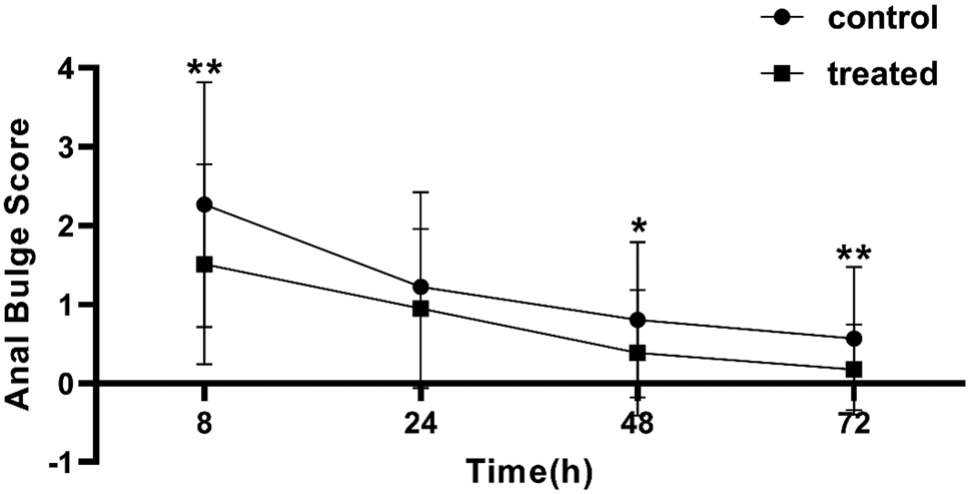
Score of anal distension within 72 h after operation. Note: *P < 0.05, **P < 0.01

The VAS scores of the patients in the EA group at 8, 24, 48, and 72 h after the operation was significantly lower than that of the control group (Fig. 3), and the anal distension scores of the patients in the EA group at 8, 48, and 72 h after the operation were also significantly lower than the control group. While 24 h after the operation, the anal distension score of the EA group was lower than that of the control group, but there was no statistical difference (Fig. 4). The use of analgesic drugs showed that the per capita administration frequency of ketorolac tromethamine in the EA group was significantly lower than that in the control group. The number of patients requiring additional ketorolac tromethamine on the first postoperative day was also significantly lower in the EA group than in the control group (Table 3). These results demonstrated that EA at Baliao point can effectively reduce postoperative pain and anal distension in the first 3 days after PPH in patients with hemorrhoids.

**Table 3:** Analgesic drug use after operation

	Time after operation (day)	Control group (*n* = 67)	EA (*n* = 57)	*P* value
Times of ketorolac tromethamine administration, mean ± SD	/	2.40 ± 1.53	1.63 ± 1.05	< 0.01
Cases of additional use of ketorolac tromethamine (*n*)	1	17	6	< 0.05
	2	7	2	> 0.05
	3	3	0	> 0.05
Cases using Bucinnazine Hydrochloride Injection (*n*)	1	2	0	> 0.05
	2	0	0	> 0.05
	3	0	0	> 0.05

The urination score 24 h after the operation in the EA group was significantly lower than that in the control group, indicating that EA at Baliao point can effectively relieve the urination disorder and urinary retention that occurred after PPH. There was no significant difference in the frequency of defecation and the characteristics of stool between the two groups on the 1st, 2nd, and 3rd days after surgery (Table 4).

**Table 4:** Defecation and urination after operation

	Time after operation (day)	Control group (*n* = 67)	EA (*n* = 57)	*P* value
First defecation time after operation, mean ± SD, h	/	28.14 ± 10.08	27.18 ± 8.82	> 0.05
Defecation frequency, mean ± SD	1	0.48 ± 0.73	0.63 ± 0.84	> 0.05
	2	1.04 ± 0.64	0.98 ± 0.55	> 0.05
	3	0.97 ± 0.30	0.89 ± 0.31	> 0.05
Bristol Stool Scale, mean ± SD	1	4.91 ± 0.73	4.92 ± 0.65	> 0.05
	2	4.55 ± 0.78	4.29 ± 0.50	> 0.05
	3	4.27 ± 0.51	4.18 ± 0.39	> 0.05
Cases of tenesmus	1	12	4	> 0.05
	2	2	0	> 0.05
	3	0	0	> 0.05
Urination score, mean ± SD	1	0.99 ± 1.65	0.38 ± 1.10	< 0.05

### Postoperative adverse events

One case of postoperative bleeding, one case of postoperative surgical incision infection and one case of reoperation due to bleeding occurred in the control group, and one case of surgical incision infection occurred in the EA group. There was no statistically significant difference between the two groups in postoperative bleeding, postoperative wound infection, and reoperation rates (Table 5). However, the number of urinary retention cases in the EA group (1 case) was significantly lower than that in the control group (5 cases), which suggested that the EA Baliao point treatment could prevent the occurrence of urinary retention in patients with hemorrhoids after PPH (Table 5).

**Table 5:** Postoperative adverse events

Group	Control (*n* = 67)	EA (*n* = 57)	*P* value
Bleeding (*n*)	1	0	> 0.05
Urinary retention (*n*)	5	1	< 0.05
Surgical incision infection (*n*)	1	1	> 0.05
Reoperation (*n*)	1	0	> 0.05

The telephone follow-up results at 1 month after operation showed that there was no hematochezia in two groups. A small number of patients in the two groups had recurrent anal pain or distension, tenesmus, constipation, or diarrhea, but there was no significant difference between the two groups. It was worth noting that some patients in both groups complained that they could not completely empty feces. The number of patients in the control group was slightly more than that in the EA group, but the difference was not statistically significant (Table 6).

**Table 6:** Follow-up outcomes at 1 month after operation

Group	Control (*n* = 67)	EA (*n* = 57)	*P* value
Anal pain or distension (*n*)	3	2	> 0.05
Hematochezia (*n*)	0	0	> 0.05
Tenesmus (*n*)	5	3	> 0.05
Constipation or diarrhea (*n*)	2	3	> 0.05
Inability to empty feces completely (*n*)	4	2	> 0.05

## Discussion

PPH surgery is a good choice for the treatment of hemorrhoids, which has the advantages of short operation time, low incidence of surgical complications, rapid postoperative recovery, and reliable surgical effect [1]. However, many studies have reported that PPH surgery is highly associated with short-term postoperative pain, tenesmus, anal distension, and urination disorders [14, 15]. In order to treat these short-term complications after PPH, various methods have been tried, but satisfactory results have not been achieved.

Acupuncture and moxibustion is a kind of treatment in traditional Chinese medicine, which has been increasingly used for perioperative treatment in recent years [16]. Several previous studies have shown that acupuncture at Baliao point can relieve pain after anorectal surgery and gynecological surgery, as well as spasm of urethral sphincter and bladder detrusor after surgery. Wang et al. reported that EA at Baliao point can treat bladder detrusor overactivity after stroke [6]. Sheng et al. also showed that EA at Baliao point can treat urinary retention caused by spinal cord injury [17]. Another study from Beijing, China, also demonstrated that acupuncture at Baliao point can promote the recovery of bladder function in patients after radical hysterectomy [18]. Su et al. showed that EA at Baliao point could promote the discharge of intrauterine residues and the recovery of uterine volume after uterine curettage [10]. Ni et al. reported that preoperative EA at Baliao point could alleviate anal pain and distension in patients with hemorrhoids at 6 h, 12 h, and 18 h after operation [13]. Unfortunately, there are some problems in the study of Ni et al., such as small sample size, insufficient postoperative observation time, and insufficient comprehensive observation indicators. Therefore, we hope to conduct a prospective clinical study with single disease, moderate sample size, and more comprehensive observation indexes through our study to further explore the effect of EA at Baliao point on the recovery of hemorrhoid patients after PPH.

A total of 124 patients with hemorrhoids who underwent PPH at Shanghai Fifth People’s Hospital were included in our study. The results showed that EA at Baliao could significantly relieve patients’ anal pain, anal distension, and postoperative urination disorder within 3 days after operation, which was consistent with the previous clinical results on EA at Baliao point. This intervention could have significant clinical relevance since post-operative pain and anal distension are common complications after PPH and can negatively impact patient recovery and quality of life. By providing a non-pharmacological alternative for pain relief, EA at Baliao point may offer a valuable addition to the current standard of care for patients undergoing PPH. It is worth mentioning that the acupoints selected in our study are secondary liao and middle liao, which are different from those reported previously. In addition to PPH, spinal anesthesia will also lead to short-term complications such as local pain and urinary retention; our EA treatment may also has therapeutic effect on local pain and postoperative urinary retention caused by spinal anesthesia and promotes the overall recovery of patients after PPH. Interestingly, there was no statistically significant difference in stool frequency and stool characteristics between the EA group and the control group within 3 days after the operation, suggesting that the effect of EA at Baliao point on defecation function and stool formation needs further research and exploration.

Postoperative complications such as postoperative infection and urinary retention occurred in both groups of patients. There was no significant difference in the incidence of postoperative infection between the two groups. While the incidence of urinary retention in the control group was significantly higher than that in the EA group, indicating that EA at Baliao point can promote the recovery of bladder function in patients, promote urine excretion, and avoid the use of catheters after PPH surgery. In addition, one patient in the control group developed severe postoperative bleeding and underwent a second operation to stop the bleeding. In the EA group, there was no postoperative bleeding or reoperation patients in the EA group. We believe that this difference may be caused by accidental factors and is not related to the EA at Baliao point.

One-month follow-up results showed that some patients in both groups had symptoms of chronic anal pain, anal bulge, tenesmus, constipation, or diarrhea, but there was no significant difference between the two groups. In both groups, some patients complained that they could not completely empty the feces during defecation, which is a common problem after PPH. The number of patients in the EA group suffering from this problem was slightly less than that in the control group, but the difference was not statistically significant. And this study also has some limitations. Firstly, whether EA at Baliao point can improve the problem of inability to completely empty feces in patients after PPH requires further research. Secondly, there is no objective laboratory index for the patients’ subjective feeling of heaving and pain. How to avoid strong subjectivity and objectively and correctly evaluate the degree of anal distension and pain after PPH needs further research and demonstration. Thirdly, although some studies have shown that the physiological mechanism of acupuncture is related to purinergic signaling, the endorphins theory [19, 20], we must admit that the mechanism of acupuncture on local acupoint stimulation is not fully understood at present, and the mechanism of EA at Baliao point to improve pelvic floor function after PPH needs further study.

## Conclusion

Our study showed that EA at Baliao point can alleviate short-term anal pain, anal distension, and urination disorders and reduce the use of analgesics in patients with hemorrhoids after PPH. Further research is needed to fully evaluate the clinical significance of this intervention and its potential use in routine clinical practice.

## Data Availability

The data used to support the findings of this study are available from the corresponding author upon request. The data are not publicly available due to privacy or ethical restrictions.
